# Difficult-to-treat rheumatoid arthritis: Current concept and unsolved problems

**DOI:** 10.3389/fmed.2022.1049875

**Published:** 2022-10-24

**Authors:** Ryu Watanabe, Tadashi Okano, Takaho Gon, Naofumi Yoshida, Kazuo Fukumoto, Shinsuke Yamada, Motomu Hashimoto

**Affiliations:** ^1^Department of Clinical Immunology, Osaka Metropolitan University Graduate School of Medicine, Osaka, Japan; ^2^Department of Orthopaedic Surgery, Osaka Metropolitan University Graduate School of Medicine, Osaka, Japan

**Keywords:** difficult-to-treat, disease-modifying antirheumatic drugs, drug resistance, methotrexate, pulmonary involvement

## Abstract

Over the past several decades, the treatment of rheumatoid arthritis (RA) has advanced significantly, and clinical, structural, and functional remission are achievable therapeutic goals. However, a substantial number of patients show resistance to multiple drugs. In particular, patients whose disease activity cannot be controlled despite the use of two or more biological disease-modifying antirheumatic drugs (DMARDs) or targeted synthetic DMARDs (tsDMARDs) with different mechanisms of action (MOA) have recently been referred to as having difficult-to-treat RA (D2T RA). D2T RA is a heterogeneous and multifactorial disease state, and the major problems are uncontrolled disease activity and decreased quality of life, as well as the economic burden due to frequent healthcare utilization and multiple admissions. Since the concept of D2T RA is relatively new and publication regarding D2T RA is limited, the mechanism underlying DMARD inefficacy and which factors form a “difficult-to-treat” state in such patients are not yet fully understood. It is also possible that factors contributing to D2T RA may differ by patient, sex, country, and race. The present Mini Review introduces the current concept and unsolved problems of D2T RA, including the definition, prevalence, and factors contributing to D2T RA. We then discuss the management and therapeutic strategies for D2T RA. Finally, we explore a clinical approach to prevent patients from developing D2T RA.

## Introduction

Rheumatoid arthritis (RA) is characterized by progressive joint destruction and impaired physical function (1). Over the last two decades, significant advances have been made in the treatment of RA. First, the revision of the classification criteria for RA in 2010 enabled early diagnosis (2). Second, the “treat-to-target strategy” has been widely accepted in which treatment is carried out toward therapeutic goals that are predefined in each patient (3). Third, biological disease-modifying antirheumatic drugs (bDMARDs) and targeted synthetic DMARDs (tsDMARDs) have become available for patients who show an inadequate response to methotrexate (MTX) (4). Lastly, evidence-based treatment recommendations for the management of RA have been proposed by the European League Against Rheumatism (EULAR) and updated every 3 years (4). These factors have made the cessation of progression of joint destruction a feasible treatment goal.

However, a substantial number of patients exhibit resistance to multiple drugs (5). In particular, patients whose disease activity cannot be controlled even with the use of two or more bDMARDs or tsDMARDs (b/tsDMARDs) with different mechanisms of action (MOA) are referred to as bearing “difficult-to-treat RA” (D2T RA) (6). D2T RA is currently considered a heterogeneous and multifactorial disease state (7, 8). The major problem with D2T RA is uncontrolled disease activity and decreased quality of life, as well as the economic burden due to frequent healthcare utilization and multiple admissions (9).

In addition, publications on D2T RA are limited because the concept of D2T RA is relatively new. The term D2T RA was proposed for the first time in 2018 (10), and the definition of D2T RA was proposed in 2021 (6). Therefore, the mechanism underlying DMARD inefficacy and which factors form a “difficult-to-treat” state in such patients are not yet fully understood (7).

This Mini Review first explains the current concept and unsolved problems of D2T RA, including the definition, prevalence, and factors contributing to D2T RA. We then discuss the management and therapeutic strategies for D2T RA. Based on these findings, we explore a clinical approach to prevent patients from developing D2T RA.

## Current status and unsolved problems of difficult-to-treat RA

### Definition of difficult*-to-treat RA*

**FIGURE 1:**
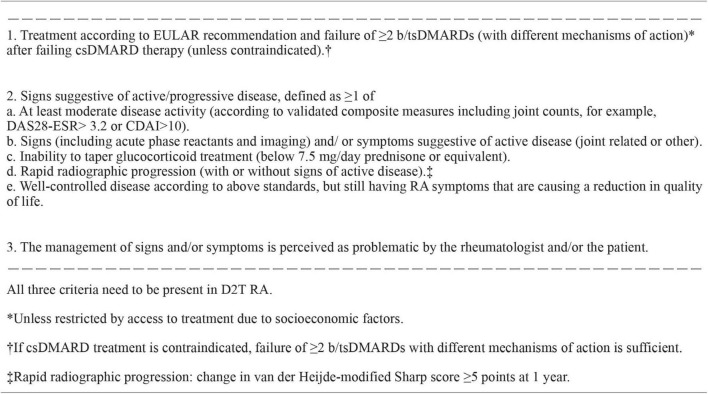
EULAR definition of difficult-to-treat RA. b/tsDMARDs, biological or targeted synthetic disease-modifying antirheumatic drug; CDAI, clinical disease activity index; CS, conventional synthetic; DAS28-ESR, disease activity score assessing 28 joints using erythrocyte sedimentation rate; D2T RA, difficult-to-treat rheumatoid arthritis; EULAR, European League Against Rheumatism; RA, rheumatoid arthritis.

In 2018, the definition of D2T RA was proposed to consist of persistency of signs and/or symptoms suggestive of inflammatory RA disease activity, despite prior treatment with conventional synthetic DMARDs (csDMARDs) and at least two bDMARDs (10). However, the choice of this cutoff bDMARDs number was arbitrary, and tsDMARDs were not included. An international survey was conducted among rheumatologists with multiple questions regarding the clinical characteristics of D2T RA. The survey demonstrated that among 410 respondents, 62% selected failure to ≥ 2 b/tsDMARDs as characteristic of D2T RA (11). Based on these results, the EULAR Task Force defined D2T RA as signs suggestive of active/progressive disease after failing csDMARD therapy and ≥ 2 b/tsDMARDs with different MOA (6) (Figure 1). Signs suggestive of active/progressive disease include (1) at least moderate disease activity (MDA) according to validated composite measures, (2) signs (including acute phase reactants and imaging) and/or symptoms suggestive of active disease, (3) inability to taper glucocorticoid treatment (below 7.5 mg/day prednisone or equivalent), (4) rapid radiographic progression on X-ray, and (5) well-controlled disease according to the above standards, but still having RA symptoms that are causing a reduction in quality of life (Figure 1). Perceptions of problems by rheumatologists and/or patients were also included. Although a variety of terms have been used to describe this patient population, including severe, refractory, multidrug/treatment resistant, and complex RA, the terminology D2T RA has been widely accepted by this definition.

However, this definition was developed based on the international survey and may not be based on sufficient evidence because high-quality studies remain scarce regarding the diagnosis of D2T RA (12). A retrospective analysis of 3,535 patients from Japan suggested that D2T RA should be defined when disease activity is not controlled with ≥ 2 csDMARDs and/or ≥ 4 b/tsDMARDs (13). A new definition and/or biomarker of D2T RA based on sufficient evidence may be necessary in the future.

### Prevalence of difficult-to-treat RA

The prevalence of D2T RA was initially estimated to be 3–10% in all RA patients (10). The international survey estimated the prevalence of 5–20% based on the British Rheumatology Biologics Registry data (11, 14). Subsequently, the proportion of patients who fulfill the criteria of D2T RA or who show multiple b/tsDMARD resistance in patients with RA has been reported in several cohort studies worldwide, mostly in the 5–20% range (15–19). In our cohort, 7.9% of 672 patients with RA fulfilled the definition of D2T RA (20). These results may reflect the widespread use of high-dose MTX and b/tsDMARDs following the treat-to-target strategy, resulting in relatively favorable disease control in 80–90% of patients with RA.

However, the problem is how to handle patients with limited access to expensive DMARDs. The reported prevalence of D2T RA in the aforementioned cohort studies mostly did not include these patients. For example, in our cohort, 21.2% showed moderate to high disease activity at the last visit, with no history of b/tsDMARD use. This population may have included patients with limited access to b/tsDMARDs owing to high costs (20). In the definition of D2T RA, the following is described in the footnote: failure of ≥ 2 b/tsDMARDs unless restricted by access to treatment due to socioeconomic factors (6) (Figure 1). Thus, D2T RA includes patients whose disease activity cannot be controlled because of limited access to b/tsDMARDs due to socioeconomic reasons. However, this may still be a point of controversy, as patients who have actually used two or more b/tsDMARDs with different MOA are inherently different from those who have not. In addition, limited access to b/tsDMARDs may be not only due to the high cost but also to physicians’ decisions because of multimorbidity and/or severe organ damage, which may be the most typical “difficult-to-treat” states. Recent studies have revealed that RA patients experience multimorbidity with an odds ratio more than twice that of non-RA patients (21, 22). Therefore, the true incidence of D2T RA cannot be determined based on the current definition of D2T RA.

### Factors contributing to difficult-to-treat RA

As described above, D2T RA is a heterogeneous and multifactorial disease state but can be largely divided into persistent inflammatory D2T RA and non-inflammatory D2T RA with little objective inflammation (5). A diverse range of factors contribute to both inflammatory and non-inflammatory D2T RA. In the initial proposal of the D2T RA concept, immunologic mechanisms underlying synovitis, smoking, drug pharmacogenesis, immunogenicity of bDMARDs, recurrent adverse drug reactions, comorbidities, negative disease outcomes such as secondary fibromyalgia, medication non-adherence, and inappropriate medication use were considered to contribute to D2T RA (10). The international survey demonstrated that, among comorbidities, rheumatology experts considered cardiovascular disease, extra-articular manifestations, and infection as the top three determinants of the development of D2T RA, followed by malignancy, diabetes mellitus, osteoporosis, pain syndrome, lung and kidney disease, depression, obesity, gastrointestinal disease, osteoarthritis, and others (11). However, as evident, it is not clear what is critical among these factors.

Therefore, we investigated the results of the cohort studies to determine the exact contribution of D2T RA in real-world clinical practice. Takanashi et al. reported from Japan that of 1,709 patients with RA, 173 (10.1%) met the criteria for D2T RA, and at the last observation, D2T RA patients were more likely to be female, seropositive, be receiving glucocorticoids, and have lung disease than non-D2T RA patients (18). Roodenrijs et al. reported from Netherlands that multiple factors were independently associated with D2T RA, such as higher disease activity, comorbidities, concomitant fibromyalgia, and obesity (17). In our previous study, D2T RA patients had significantly higher rheumatoid factor (RF) levels, higher disease activities, more impaired physical functions, and a higher prevalence of lung and neurological diseases at baseline than non-D2T RA patients. In a multivariate analysis, high RF levels, high disease activity at baseline, and coexisting pulmonary disease were associated with D2T RA (20).

If not only D2T RA patients but also patients who show multiple resistance to bDMARDs were taken into consideration, the British Rheumatology Biologics Registry demonstrated that baseline factors associated with multiple resistance to bDMARDs included women, higher disease activity, current smokers, obesity, and greater social deprivation (14). Novella-Navarro et al. reported that higher baseline disease activity, the presence of erosions at baseline, and the absence of clinical response to the first bDMARDs were independently associated with multiple failures of bDMARDs (15). Moreover, a recent systematic review and meta-analysis revealed that old age, female sex, smoking history, obesity, poor functional status, high disease activity, and elevated erythrocyte sedimentation rate were poor predictors of remission with bDMARDs (23).

**FIGURE 2:**
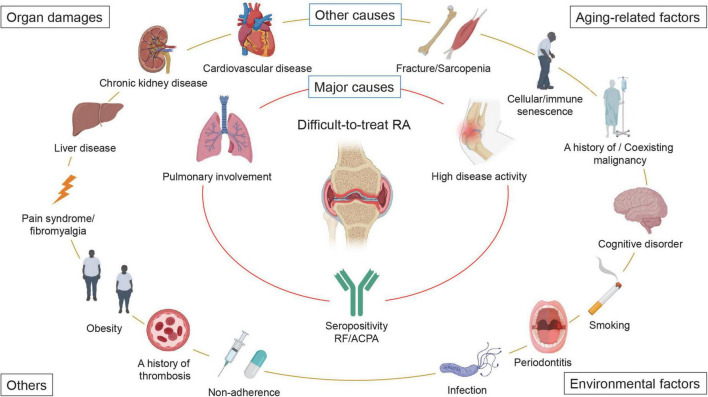
Factors that contribute to the development of difficult-to-treat RA. A wide variety of factors contribute to difficult-to-treat rheumatoid arthritis (D2T RA). We propose that high baseline disease activity, seropositivity for rheumatoid factor (RF) and anti-cyclic citrullinated peptide antibody (ACPA), and pulmonary involvement are the major causes of D2T RA, particularly in Asian countries. In addition, (1) aging-related factors, such as fracture/sarcopenia, cellular/immune senescence, a history of/coexisting malignancy, and cognitive disorders; (2) organ damage, such as cardiovascular disease, chronic kidney disease, liver disease, and pain syndrome/fibromyalgia; (3) environmental factors, such as smoking, periodontitis, infectious diseases; and (4) other factors, such as obesity, non-adherence, and a history of thrombosis also participate in the development of D2T RA. It should be noted that these factors may vary by patient, sex, country, and race.

Based on the theoretical factors that can form D2T RA and the results of cohort studies, we propose that high disease activity at baseline, seropositivity for RF and anti-cyclic citrullinated peptide antibody (ACPA), and pulmonary involvement are the major causes of D2T RA, particularly in Asian countries (Figure 2). In addition, (1) aging-related factors, such as fracture/sarcopenia, cellular/immune senescence, a history of/coexisting malignancy, and cognitive disorders; (2) organ damage, such as cardiovascular disease, chronic kidney disease, liver disease, and pain syndrome/fibromyalgia; (3) environmental factors, such as smoking, periodontitis, infectious diseases; and (4) other factors, such as obesity, non-adherence, and a history of thrombosis also participate in the formation of D2T RA (Figure 2). However, it should be noted that these factors may vary by patient, sex, country, and race.

## Management and therapeutic strategies for difficult-to-treat RA

The EULAR Task Force proposed several points that should be considered when encountering patients with D2T RA (24). Briefly, the possibility of misdiagnosis and/or the presence of a coexisting mimicking disease should be considered as the first step. Then, reassessment of comorbidities that mimic signs and/or symptoms of arthritis as well as disease activity using ultrasonography should be performed. Treatment adherence should also be discussed. Then, we consider increasing the dose of the current drug or switching a b/tsDMARD with different MOA. Additionally, non-pharmacological interventions such as exercise, education, support, and self-management programs should be considered.

Although there is insufficient evidence to determine which b/tsDMARDs are best to switch to when switching drugs, a retrospective analysis of 2,128 cases demonstrated that among tumor necrosis factor (TNF) inhibitors, IL-6 inhibitors, T cell co-stimulatory signal inhibitors, and Janus kinase (JAK) inhibitors, JAK inhibitors showed the most favorable outcomes, such as remission induction rates (19). Compared to TNF inhibitors, IL-6 inhibitors, and T-cell co-stimulatory signal inhibitors had similar effects on improving disease activity and physical function, but JAK inhibitors were significantly more effective than TNF inhibitors in such outcomes. In our preliminary cohort study, drug retention was significantly higher in patients treated with JAK inhibitors or IL-6 inhibitors than in those treated with TNF inhibitors or T cell co-stimulatory signal inhibitors. Multivariate analysis using Cox proportional hazard models demonstrated that discontinuation of the drug was associated with the use of TNF inhibitors, T cell co-stimulatory signal inhibitors, and concomitant glucocorticoid use (25). Another study by Yoshii et al. also demonstrated that concomitant use of glucocorticoids was associated with treatment failure in patients with D2T RA (26). Further studies are needed to establish additional evidence regarding how pharmacological and non-pharmacological interventions should be combined to treat D2T RA (27).

## Discussion: How to prevent patients from becoming difficult-to-treat RA?

### Early identification of patients who develop difficult-to-treat RA

As we have seen thus far, D2T RA is a highly heterogeneous patient population associated with various contributing factors. As the treatment of D2T RA is not straightforward and requires a combination of pharmacological and non-pharmacological interventions, the question is whether we can identify patients at risk of developing D2T RA and eventually prevent them from becoming D2T RA.

Several attempts have been made to identify patients at risk for developing D2T RA at baseline or during the early treatment phase. Messelink et al. reported several important features to identify D2T RA patients based on logistic regression analysis, which included the time from RA diagnosis, disease activity, and the number of prior medications (16). Another study by Becede et al. demonstrated that female sex, delay in initial treatment, and higher disease activity were predictors of a later refractory course (28). In our study, patients who had (1) high RF levels (RF of ≥ 156.4 IU/mL), (2) high disease activity at baseline, and (3) coexisting pulmonary disease were at high risk for developing D2T RA (20). Thus, D2T RA may most likely occur if treatment is delayed in patients with high disease activity and comorbidities, such as pulmonary disease.

### Therapeutic strategies to prevent patients from becoming difficult-to-treat RA

Since multiple studies have reported that the time from diagnosis to initiation of treatment is closely related to the onset of D2T RA (16, 18, 28), early intervention with b/tsDMARDs is crucial, particularly for highly active patients who show inadequate response to high-dose MTX. We believe that early intervention with b/tsDMARDs is important for suppressing not only joint destruction (4, 29), but also the onset of D2T RA. Selection of an appropriate b/tsDMARD is also critical for obtaining therapeutic effects at an early treatment course. In recent years, there have been increased reports on predicting optimal b/tsDMARDs using various technological platforms, such as machine learning and multi-omics analyses (30–33). Further studies are needed on the risk factors and treatment strategies for D2T RA, using genomic information and synovial tissue samples, with an in-depth consideration of the pathogenesis of the disease.

Additionally, among the various contributing factors for D2T RA, some are intervenable, such as smoking, obesity, poor adherence, and periodontitis, whereas others are difficult to intervene with, such as irreversible organ damage. Correcting intervenable factors is also important for suppressing the development of D2T RA. Moreover, patients with RA should have a chance to access non-pharmacological therapy, such as appropriate exercise, education, and support, early after diagnosis.

Lastly, since patients in remission have significantly better quality of life, and functional and structural outcomes than those with low disease activity, the primary goal of RA should be remission (34). Therefore, we may need to consider treatment aimed at remission in all patients with RA, not just suppression of the onset of D2T RA.

In conclusion, D2T RA is a complex condition and various factors contribute to its development. To prevent the onset of D2T RA, it is critical to treat patients with appropriate drugs early after diagnosis, while appropriately evaluating the status of comorbidities and environmental factors.

## Author contributions

RW drafted the manuscript. TO, TG, NY, KF, SY, and MH revised and finalized the manuscript. All authors read and approved the final version of the manuscript.
